# Transcriptomics of CD29^+^/CD44^+^ cells isolated from hPSC retinal organoids reveals a single cell population with retinal progenitor and Müller glia characteristics

**DOI:** 10.1038/s41598-023-32058-w

**Published:** 2023-03-28

**Authors:** Karen Eastlake, Joshua Luis, Weixin Wang, William Lamb, Peng T. Khaw, G. Astrid Limb

**Affiliations:** NIHR Biomedical Research Centre at Moorfields Eye Hospital, UCL Institute of Ophthalmology, 11-43 Bath Street, London, EC1V 9EL UK

**Keywords:** Glial biology, Stem cells in the nervous system, Cell biology, Stem cells, Retina

## Abstract

Müller glia play very important and diverse roles in retinal homeostasis and disease. Although much is known of the physiological and morphological properties of mammalian Müller glia, there is still the need to further understand the profile of these cells during human retinal development. Using human embryonic stem cell-derived retinal organoids, we investigated the transcriptomic profiles of CD29^+^/CD44^+^ cells isolated from early and late stages of organoid development. Data showed that these cells express classic markers of retinal progenitors and Müller glia, including *NFIX*, *RAX*, *PAX6*, *VSX2*, *HES1*, *WNT2B*, *SOX*, *NR2F1*/*2*, *ASCL1* and *VIM*, as early as days 10–20 after initiation of retinal differentiation. Expression of genes upregulated in CD29^+^/CD44^+^ cells isolated at later stages of organoid development (days 50–90), including *NEUROG1*, *VSX2* and *ASCL1* were gradually increased as retinal organoid maturation progressed. Based on the current observations that CD24^+^/CD44^+^ cells share the characteristics of early and late-stage retinal progenitors as well as of mature Müller glia, we propose that these cells constitute a single cell population that upon exposure to developmental cues regulates its gene expression to adapt to functions exerted by Müller glia in the postnatal and mature retina.

## Introduction

Müller glia are radial glial cells that expand across all neural layers of the retina. Anatomically, they extend characteristic filopodial processes into the different synaptic layers, facilitating their close interaction with all retinal neurons. This structural pattern allows Müller glia to perform a wide range of roles that are important for normal retinal function, including structural configuration, ion homeostasis, neurotransmitter recycling, and light processing^1–3^.

It is well documented that adult zebrafish possess the ability to regenerate the retina and that in response to injury, Müller cells de-differentiate and re-acquire phenotypic and molecular characteristics of multipotent retinal progenitors, including activation of Notch-Delta signalling, and expression of *rx1*, *pax6a* and vsx2/*chx10*^4^. In this species, retinal progenitors in turn give rise to new neurons to efficiently replace cells lost to injury^4–6^. regenerative functions of Müller glia have also been identified in avian and mammalian species during early postnatal life, including chick^7^, rat^8^ and mouse^9^, as well as in adult primate retinal explants in vitro^10^. Interestingly, Müller glia in the adult human retina also express markers of neural progenitors, such as *SOX2*, *PAX6 and VSX2* (CHX10) and a population of these cells has been shown to proliferate indefinitely in vitro^11,12^. Although human Müller glia can be induced to differentiate into cells expressing markers of retinal neurons in vitro^12^, there is no evidence that these cells have in vivo regenerative ability. Instead, as a result of injury or disease, Müller glia undergo structural and molecular changes that lead to gliosis and degeneration^13^. Therefore, understanding the molecular mechanisms of Müller glia proliferation and differentiation during development may aid in the design of protocols to promote mechanisms of retinal regeneration after disease on injury in the adult human eye.

It is widely documented that during embryonic development, Müller glia arise from the same multipotent progenitor that gives rise to all retinal neurons^14^, and it is of interest that most developmental studies have identified Müller glia as being one of the last-born cells that populate the neural retina^14–16^. However, based on historical reports, it is important to take into account that earlier light and transmission electron microscopy studies identified the presence of Müller glia during early and mid-gestational development in the chick, rat, rabbit and cat. Early ultrastructural microscopy studies in the chick retina at embryonic days 3–4 (E3–4) showed the early emerging Müller glia, characterized by their columnar assembly and nuclei predominantly accumulating near the vitreal surface^17^. Other studies showed that in the rat, soon after the optic cup invagination is completed, the innermost retinal layer exhibits Müller cell processes emerging before initiation of retinal ganglion cell axon formation^18,19^. Evidence has also been presented that the inner limiting membrane consisting of the basal lamina and foot processes of Müller glia have already formed in retinal tissue obtained during the mid gestation period in the cat (day E36)^20^ and rabbit (day E16)^21^. In the rabbit at day E25, radially oriented cells were observed staining weakly for vimentin, but at day E28 this staining became stronger^22^, indicating the presence of Müller glia.

More recent transcriptomic studies have reported expression of traditionally known markers of retinal progenitors and Müller glia in the mature retina of rodents^23,24^ and humans^25–27^. In addition, single cell RNA sequencing of cells isolated from retinal organoids derived from rodent and human pluripotent stem cells, have shown a wide range of genes previously reported in progenitor cells^25,28–30^. Taking into consideration that markers used to identify Müller glia for single cell analysis, such as CRALPB and Kir4, are in fact markers of mature and functional retina, there is very little overlap between different studies with respect to the genes expressed by these cells, with traditional markers of ‘retinal progenitors’ including *RLPP1*, *NFIB*, *NFIA*, *NFIX*, *DKK3*, *and VSX2*, and other Müller glia markers, such as *VIM* and *AQP4*, frequently found enriched in these sequencing studies. These findings are therefore in agreement with previous transcriptomic reports on the developing vertebrate retina that have identified striking similarities between Müller glia and multipotent late retinal progenitors (RPC)^23,31,32^.

Our previous studies on retinal organoids have identified a population of CD29^+^/CD44^+^ cells that are arranged in a columnar form at very early stages of organoid development and histologically resemble Müller glia. Upon isolation from 70 to 90 day old retinal organoids^33^, they show phenotypic and transcriptomic features of Müller glia. In view of the previously reported similarities in gene expression between progenitor cells and Müller glia, we investigated the transcriptomic profile of CD29^+^/CD44^+^ cells, isolated at different stages of organoid development (from D10 to D90), to identify whether their genotypic signature is characteristic of retinal progenitors and/or Müller cells. The results showed that the CD29^+^/CD44^+^ cell population can be isolated from retinal organoids between days 10–90 after initiation of differentiation and maintain several genetic features ascribed to both, retinal progenitors and Müller glia throughout the period of study.

## Methods

### Cell maintenance and isolation from retinal organoids

CD29^+^/CD44^+^ cells were isolated from retinal organoids derived from the human embryonic stem cell (ESC) line RC9 (Roslin Cells, Edinburgh, UK) using methods adapted from Nakano et al.^34^ as previously described^33^. Retinal organoids at different stages of development, including days 10, 20, 30, 50, 70 and 90 after initiation of retinal differentiation, were dissociated into single cells using a gentle cell dissociation reagent (Stem Cell Technologies, UK) (5 min at 37 °C), and plated onto fibronectin coated tissue culture plates (50 µg/ml) in the presence of fibroblast growth factor (FGF) (20 ng/ml) and epidermal growth factor (EGF) (20 ng/ml) in DMEM containing 10%FCS and 1% penicillin–streptomycin. After 3 days in culture, cells expressing the surface markers CD29 and CD44 that had selectively adhered to fibronectin, were detached, washed and total RNA was extracted using Qiagen Rneasy micro plus kit (Cat. 74034, Qiagen, UK). For each time point three biological replicates consisting of 6–10 organoid pools were used for isolation of CD29^+^/CD44^+^ cells.

### RNA sequencing

#### Sample processing and sequencing

Full RNA processing and sequencing were conducted by the UCL Genomics department at the Institute of Child Health using the following protocols.

RNA integrity was confirmed using Agilent’s 2200 Tapestation. Samples were processed using the KAPA mRNA HyperPrep Kit (p/n KK8580) according to manufacturer’s instructions. Briefly, mRNA was isolated from total RNA using Oligo dT beads to pull down poly-adenylated transcripts. The purified mRNA was fragmented using chemical hydrolysis (heat and divalent metal cation) and primed with random hexamers. Strand-specific first strand cDNA was generated using Reverse Transcriptase in the presence of Actinomycin D. This allows for RNA dependent synthesis while preventing spurious DNA-dependent synthesis. The second cDNA strand was synthesised using dUTP in place of dTTP, to mark the second strand. The resultant cDNA was then “A-tailed” at the 3’ end to prevent self-ligation and adapter dimerisation. Full length xGen adaptors (IDT), containing two unique 8 bp sample specific indexes, a unique molecular identifier (N8) and a T overhang were ligated to the A-Tailed cDNA. Successfully ligated cDNA molecules were then enriched with limited cycle PCR (12 cycles). The high fidelity polymerase employed in the PCR is unable to extend through uracil. This means only the first strand cDNA is amplified for sequencing, making the library strand specific (first-strand).

Libraries to be multiplexed in the same run were pooled in equimolar quantities, calculated from Qubit and Bioanalyser fragment analysis. Samples were sequenced on the HiSeq 3000 instrument (Illumina, San Diego, US) using a 75 bp paired read run with a corresponding 8 bp UMI read.

#### Data analysis

Run data were demultiplexed and converted to fastq files using Illumina’s bcl2fastq Conversion Software v2.19. Fastq files were then aligned to the human genome UCSC hg38 using RNA-STAR 2.5.2b then UMI deduplicated using Je-suite (1.2.1). Reads per transcript were counted using FeatureCounts and differential expression was estimated using the BioConductor package SARTools, a DESeq2 wrapper. The normalisation and differential expression analysis was performed by DESeq2, within a package called SARTools. The results were pre-filtered into up regulated and down regulated genes (2.5-fold change; p < 5%).

The raw RNA Seq datasets generated and analysed during the current study are available in ArrayExpress repository (https://www.ebi.ac.uk/biostudies/arrayexpress) with the accession no. E-MTAB-12467.

Bioinformatic analysis of RNA-seq data was conducted using the online platform iDEP90 (http://ge-lab.org/idep/) which conducts differential expression and pathway analysis. Analyses of read count data were conducted using iDEP 0.91, hosted at http://ge-lab.org/idep/.

### Immunohistochemistry of retinal organoids

Organoids were fixed in 4% paraformaldehyde for 5 min prior to cryoprotection in 30% sucrose for 30 min, embedded in OCT, and snap frozen on dry ice. Tissue sections were cut at 10–12um using a Leica Cryostat (CM1850) and mounted on Superfrost plus slides (VWR, UK). For antibody staining, sections were blocked for 1 h in TBS + 0.3% triton X + 5% donkey serum prior to the addition of the primary antibody diluted in the same blocking buffer. Primary antibodies were incubated overnight at 4 °C. Cells were then washed with TBS three times for 5 min. Secondary antibodies (Alexa flour, 1:500 in TBS + 0.3% triton) were incubated for 3 h at room temperature in the dark. Slides were then washed in TBS and coverslips mounted with Fluoroshield Mounting Medium containing DAPI (Abcam; ab104139), and sealed with nail varnish.

Immunohistochemistry was used to confirm retinal organoid growth over the total 90 day differentiation period. To follow the developing retinal organoids we performed IHC at the same time points that cells were isolated (D10, 20, 30, 50, 70 and 90) to identify cells expressing CD29/CD44 in-situ as well as to show other retinal cell markers to confirm differentiation stages. Immunohistochemical staining of organoids for Müller markers as well as for other retinal cell markers as shown in supplementary Figs. 1 and 2.

### Quantitative PCR

Total RNA was extracted using Qiagen Rneasy micro plus kit (Cat. 74034, Qiagen, UK). For reverse transcription, the SuperScript IV First-Strand Synthesis System (Life Technologies) was used as per manufacturer’s instructions. Briefly, 0.5 µg of oligo (dT) 12–18 primers (Life Technologies) and 0.5 mM dNTPs (Life Technologies) were incubated with 250 ng of RNA and heated to 65 °C for 5 min using an Eppendorf Mastercycler (Eppendorf). To the reaction mix, 5 mM DTT, 40U of RNasin Plus (Promega) and 200 U of Superscript IV in first strand buffer, were added and cDNA was generated by incubation at 55 °C for 10 min and heating to 80 °C for 10 min. For each reaction 2.5 ng cDNA was used. The qRT-PCR was performed using SYBR green Gene expression assays. Primers for selected genes can be found in supplementary table 1 (SI Tab 1). The thermo cycling conditions were 95 °C for 10 min followed by 40 cycles of 95 °C for 15 s and 60 °C for 1 min. The data was analysed using the 2 − ΔΔCt method as fold change relative to GAPDH as endogenous control. All samples were run in triplicates. Correlation in expression abundancies between RNA-seq and qRT-PCR was determined using the Pearson’s correlation coefficient.

## Results

### CD29^+^/CD44^+^ immunoreactivity within retinal organoids and in dissociated cells

**Figure 1:**
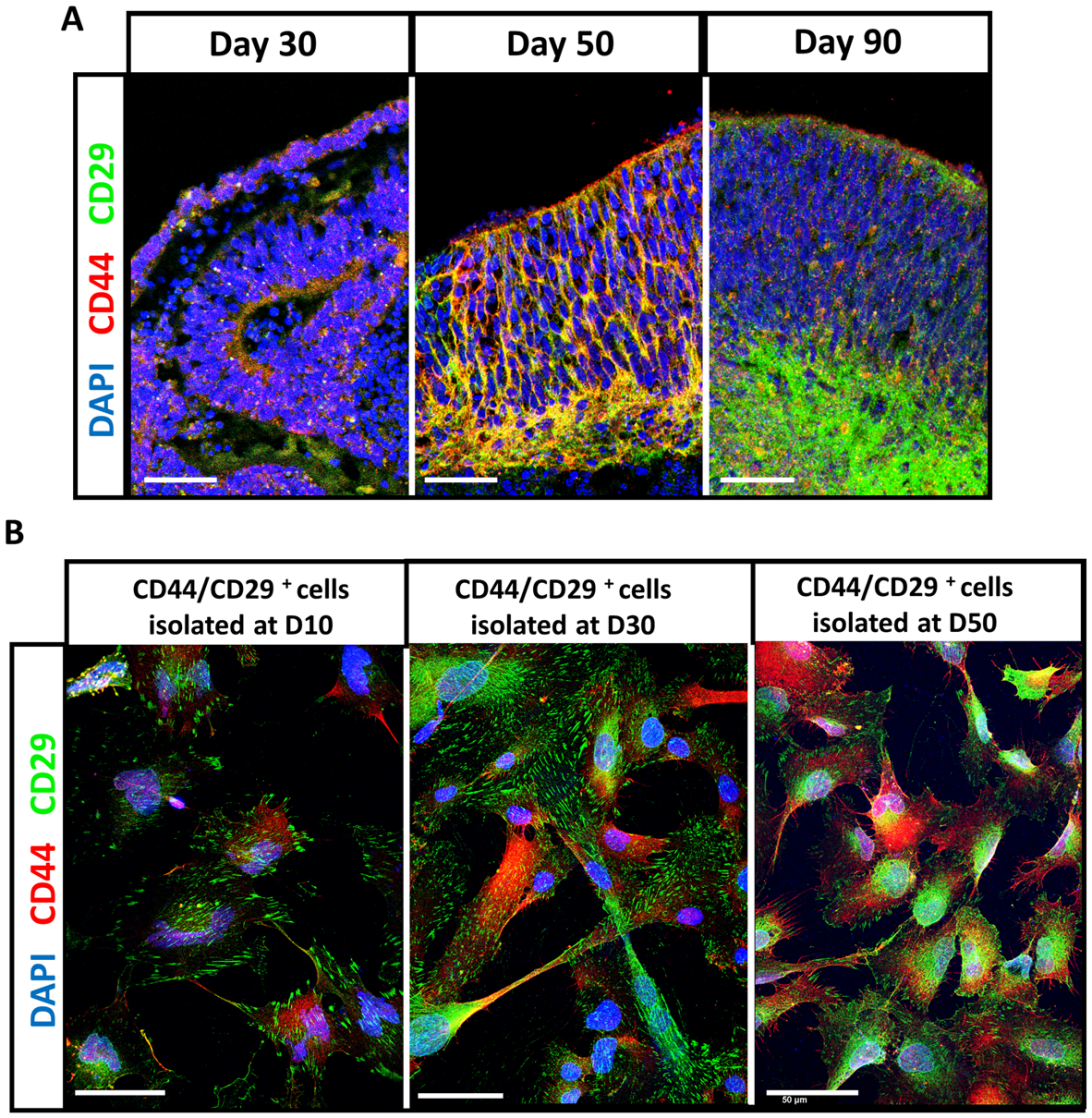
Isolation of CD29^+^/CD44^+^ cells from retinal organoids. (**A**) Confocal images show positive staining for CD29 (green) in retinal organoids from days 20–70 after initiation of differentiation. (**B**) Representative confocal images show positive staining for CD29 (green), and CD44 in CD29^+^/CD44^+^ cells isolated from day 10 (D10), 30 (D30) and day 50 (D50) organoids. Scale bar = 50 μm. Nuclei stained with DAPI (blue).

Our previous studies on retinal organoids have identified a population of cells that are arranged in a columnar form at very early stages of organoid development that resemble Müller glia. We have shown that these cells can be isolated using affinity of the cell surface receptors CD29 and CD44 for fibronectin which are characteristically expressed by Müller glia. Immunostaining of retinal organoids at developmental day (D) 30 showed expression of the well-known Müller cell markers CD29 and CD44. Rosette spheroids were also observed at this stage, where CD29 positive cells exhibit an inverse polarity. This feature is observed during re-aggregation of embryonic chick retinal cells in vitro, where Müller glia localize in the inner region and the initiation of a radial arrangement can be recognized^35^ (Fig. 1A, left image). At days 50 and 90, CD29 and CD44 positive cells resemble the distribution of Müller glia in the mature retina, in which these cells are anchored at both, the inner and outer limiting membrane^35^ (Fig. 1A, centre and right images). Co-expression of CD29 with CD44 and vimentin, was also observed in cells isolated from retinal organoids at all the stages of organoid differentiation examined (D10, 30 and D50 isolated cells shown, Fig. 1B). Furthermore, staining for known Müller and other retinal cell markers during organoid development and maturation showed distribution and polarity, characteristic of Müller glia. Cells expressing vimentin and nestin were observed clustered in the organoid borders as early as D10 of organoid differentiation. At days D20 and D30, cells expressing these Müller markers were observed extending processes throughout the organoids and their distribution was then maintained until D90. Positive staining for the photoreceptor marker Rhodopsin was not observed in the organoids until D70, whilst robust expression of recoverin was observed in D30–D90 organoids. OTX2 which is required for photoreceptor differentiation and bi-polar maturation was expressed from D10 onwards, whereas a subpopulation of OTX2 positive cells that co-stained with recoverin was observed from D30 onwards. TUJ1 staining (identifying beta III tubulin expression), expressed by RGCs was increasingly observed from D10 onwards. By D50 long beta III tubulin positive processes could be observed (Supplementary Figs. 1 and 2).

### Transcriptome of CD29^+^/CD44^+^ cells isolated from retinal organoids at different stages of maturation

**Figure 2:**
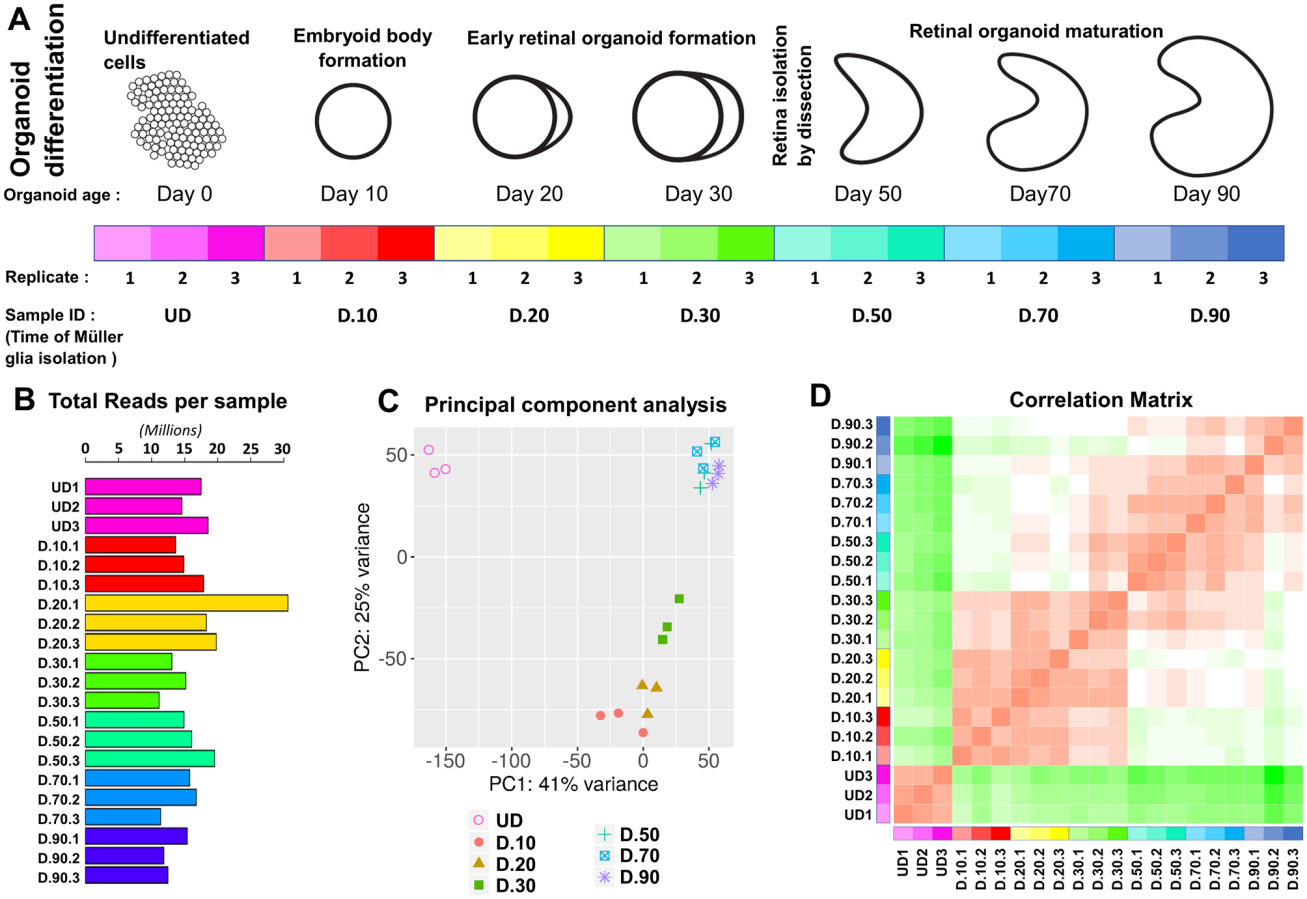
Transcriptomic analysis and overview and RNA seq libraries. (**A**) Schematic drawing shows developmental stages of retinal organoid development, and description of cells collected from each stage of development. (**B**) Bar graph shows the total number of reads obtain from each sample (**C**) principal component plot shows the clustering of each transcriptome group, including undifferentiated stem cells (UD) and Müller glia isolated from retinal organoids at days 10, 20, 30, 50, 70 and 90 after initiation of differentiation. (**D**) Matrix shows the RNA-seq correlation between samples as determined by Pearson correlation coefficients.

In this study, CD29^+^/CD44^+^ cells were isolated from retinal organoids at seven different time points after initiation of retinal organoid differentiation and processed for RNA seq analysis (Fig. 2A). Test samples included an undifferentiated stem cell control (UD), cells isolated from organoids at D10, 20, 30, 50 and 90. We obtained between 10 and 20million reads per sample (Fig. 2B), except for one replicate which yielded over 30million reads. The principal component analysis (PCA) showed close clustering of biological replicates, similar clustering of early or late stage isolated CD29^+^/CD44^+^ cells and separate clustering of the undifferentiated stem cells (Fig. 2C). The sample correlation matrix was used to assess the variance in the gene expression profile between samples and showed a close relationship between the biological replicates, and similar gene expression patterns of CD29^+^/CD44^+^ cells isolated at D10, 20, 30, 50, 70 and 90 of retinal organoid maturation. The correlation matrix also confirmed that the undifferentiated stem cells have a completely different profile to that of the isolated CD29^+^/CD44^+^ cells, to which gene expression increasingly differs as time points of cell isolation progresses (Fig. 2D).

**Figure 3:**
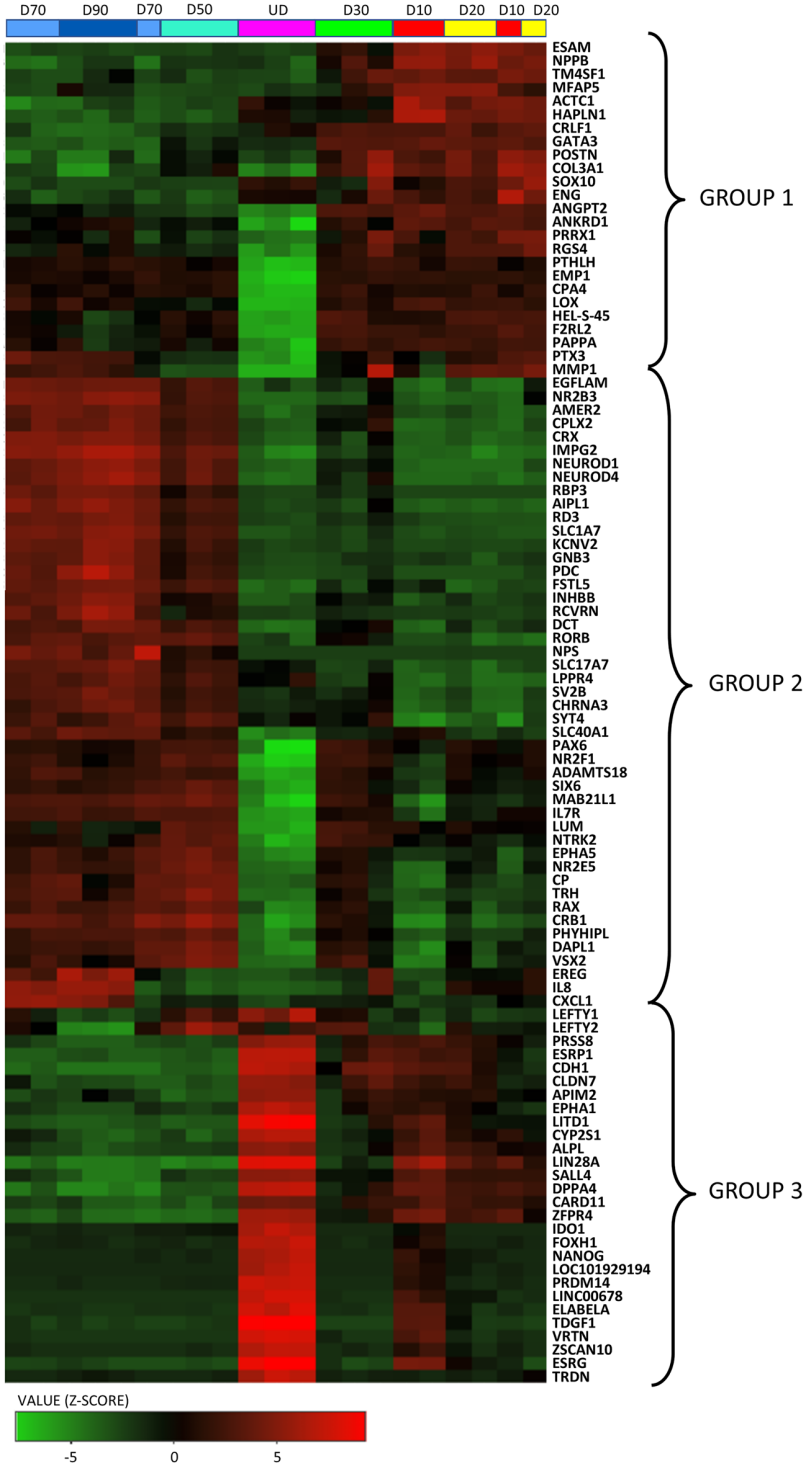
Heatmap of the top 50 variable genes. Heatmap shows the top 50 most variable genes amongst all sample replicates. All genes were ranked by standard deviation across all samples, then used in hierarchical clustering using the heatmap.2 function in iDEP91. Colour key indicates z-score (green = negative; black = no change; red = positively expressed).

Expression analysis of the top 100 most variable genes across all samples showed distinct grouping. The heatmap anlysis showed a clear difference in gene expression levels between cells isolated from organoids at days 10–30 (D10, D20, D30) and days 50–90 (D50, D70, D90) (Fig. 3). As expected,the undifferentiated stem cells (UD) showed a vastly different expression profile to that of the CD29^+^/CD44^+^ isolated cells. Gene expression patterns showed a gradual deviation from their starting material, where CD29^+^/CD44^+^ cells isolated at D10 showed some similar patterns to that of the UD cells and cells isolated from D50–D90 retinal organoids exhibited greater differences in expression from the starting material. Genes that were preferentially expressed in CD29^+^/CD44^+^ cells isolated from retinal organoids at D10–D30 as compared to the UD stem cells or those isolated from D50–D90 retinal organoids were termed ‘group 1’ (Fig. 3) and included *NPPB* (brain natriuretic peptide), *POSTN* (periostin), *SOX10*, *MMP1* and *PTX3* (pentraxin3) which have previously been shown to be expressed by Müller glial cells^36–38^. Genes shown to be upregulated in CD29^+^/CD44^+^ cells isolated from D50–D90 retinal organoids as compared to UD cells or cells isolated from D10–D30 retinal organoids were termed ‘group 2’ (Fig. 3) and included genes with known roles in Müller glia that are reportedly expressed by retinal progenitor cells, including *CRB1*, *EGFLAM* (Pikachurin), *VSX2*, *PAX6*, *RAX* and *SIX6*. Other genes in this group included, *NEUROD1*/*4* (neuronal differentiation 1 and 4), *SYT4* (synaptotagmin 4), *CRX* (cone-rod homeobox), *CRB1*, *KCNV2* (potassium v-gated channel modifier subfamily v-member 2), *PDC* (phosducin), *RBP3* (retinol binding protein3), *NR2B3*, *NR2F1* and *RCVRN* (recoverin) which are all associated with retinal development and may be related to progenitor like roles of Müller glia. Finally, genes which were termed ‘group 3’ included those which were highly expressed in the undifferentiated stem cells (Fig. 3). These genes included *NANOG*, *FOXH1*, *ESRG*, and *VTRN* which are all stem cell pluripotency related genes, and were markedly downregulated with maturation.

### Gene Ontology (GO) analysis of DEGs

We used the online tool iDEP91 (http://bioinformatics.sdstate.edu/idep/) to examine the expression patterns of isolated CD29^+^/CD44^+^ cells from retinal organoids upon increasing maturity. We identified differentially expressed genes between groups with the DESeq2 package based on a false detection rate (FDR) of < 0.1 and > 2.5-fold change in expression. Differentially expressed genes, were subject to gene ontology analysis using the parametric gene set enrichment analysis (PGSEA; Furge and Dykema, 2012) using iDEP91, to compare between all sample groups. The total differentially expressed genes, uniquely expressed genes of isolated CD29^+^/CD44^+^ cells, and pathway over-representation analysis can be found in Supplementary Fig. 3. The most striking expression of unique DEGs were found on cells isolated at Day 10 when compared with UD cells, with 1885 genes upregulated and 2188 genes downregulated. The next prominent change in unique DEGs was seen when comparing CD29^+^/CD44^+^ cells isolated between D50 and D30, when 404 genes were upregulated and 578 were downregulated. Between these time points the growing retina ‘mantle’ of the retinal organoids were dissected from the from the rest of the embryoid body to ensure that only the neural retina was left to mature in culture. These results therefore reflect retinal development and increasing maturity of the retinal organoids with extended culture (Supplementary Fig. 3).

**Figure 4:**
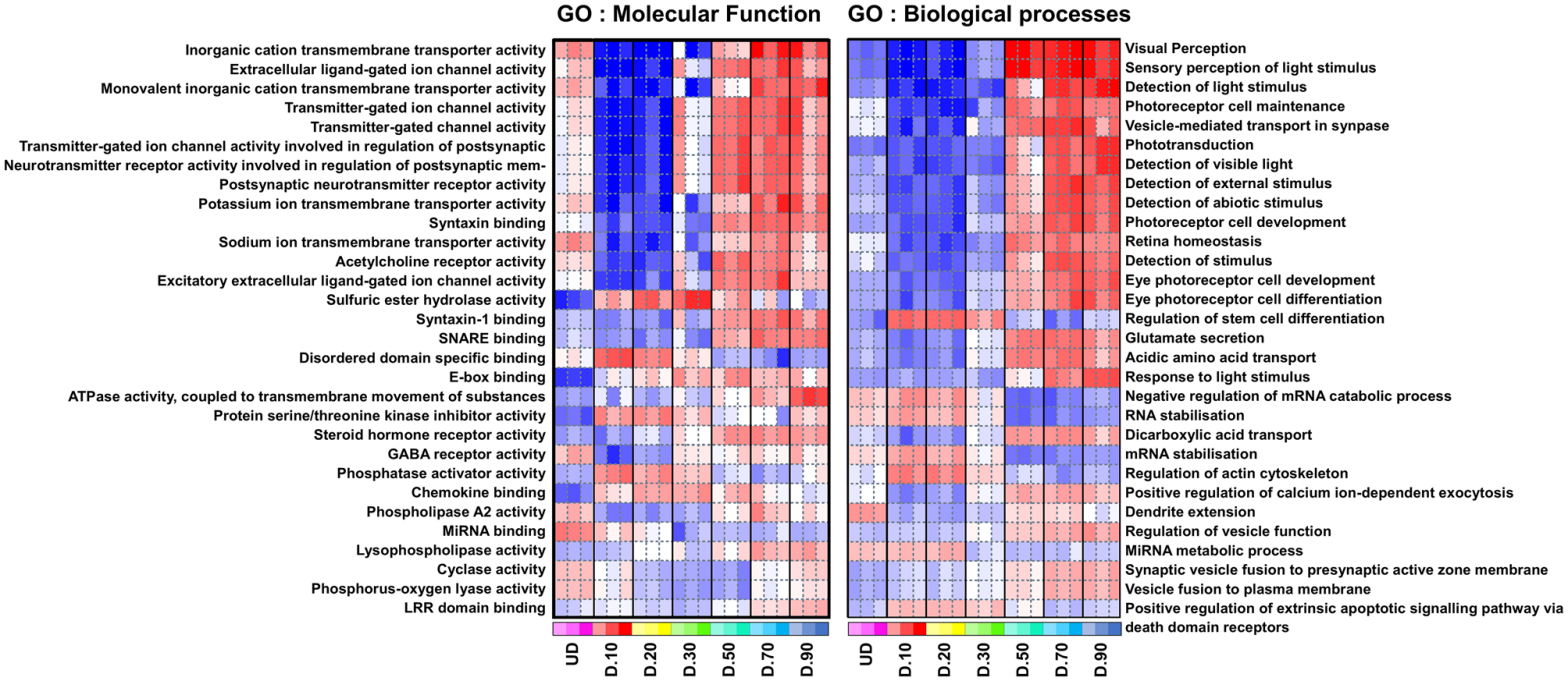
Gene Ontology Analysis of DEGs. Heatmaps illustrates gene ontology evaluation using the parametric gene set enrichment analysis (PGSEA; Furge and Dykema, 2012) in iDEP91, to compare between all sample groups. DEGs were classified into different functional categories according to the GO term enrichment analysis for (**A**) molecular function and (**B**) Biological process.

Differentially expressed genes (DEGs) were classified into different functional categories according to the GO term enrichment analysis for Biological processes and Molecular function that showed transcriptional differences between CD29^+^/CD44^+^ cells isolated at different stages of organoid differentiation (Fig. 4). The GO Molecular function annotations showed increases in various ion channel genes, syntaxin binding and ATPase activity, amongst other regulatory changes (Fig. 4). These may be ascribed to acquisition of retinal functions as more neuronal cells populate the organoid during maturation. The GO Biological process analysis also revealed changes in genes associated with eye development, visual perception, regulation of stem cell differentiation, regulation of the actin cytoskeleton, dendrite extension and metabolic processes. These processes were shown to be highly enriched in the CD29^+^/CD44^+^ cells isolated between D50 to 90 of organoid maturation as compared to the undifferentiated stem cells or cells isolated from the earlier developing organoids at D10–30.

### Examination of transcripts associated with Müller glia cell fate determination in CD29^+^/CD44^+^ cells throughout organoid development

We compared the expression of well characterised Müller glia markers in CD29^+^/CD44^+^ isolated at different stages of organoid development to assess how this profile changes during maturation. In addition, genes previously identified by others to be involved in Müller cell fate determination^39–41^ were analysed for their expression in the isolated cell populations. Using log-twofold changes in expression, we regarded a 1.32 log2 fold change (increase or decrease) the cut off for significant changes in expression, corresponding to fold changes of 2.5 and 0.4 respectively. We performed two comparision analyses to identify gene changes in the cell populations, where all isolated CD29^+^/CD44^+^ cell groups D10–D90 were compared with the undifferentiated stem cells or cell groups D20–90 were compred to D10 isolated cells with cells isolated between D20 to D90.

**Figure 5:**
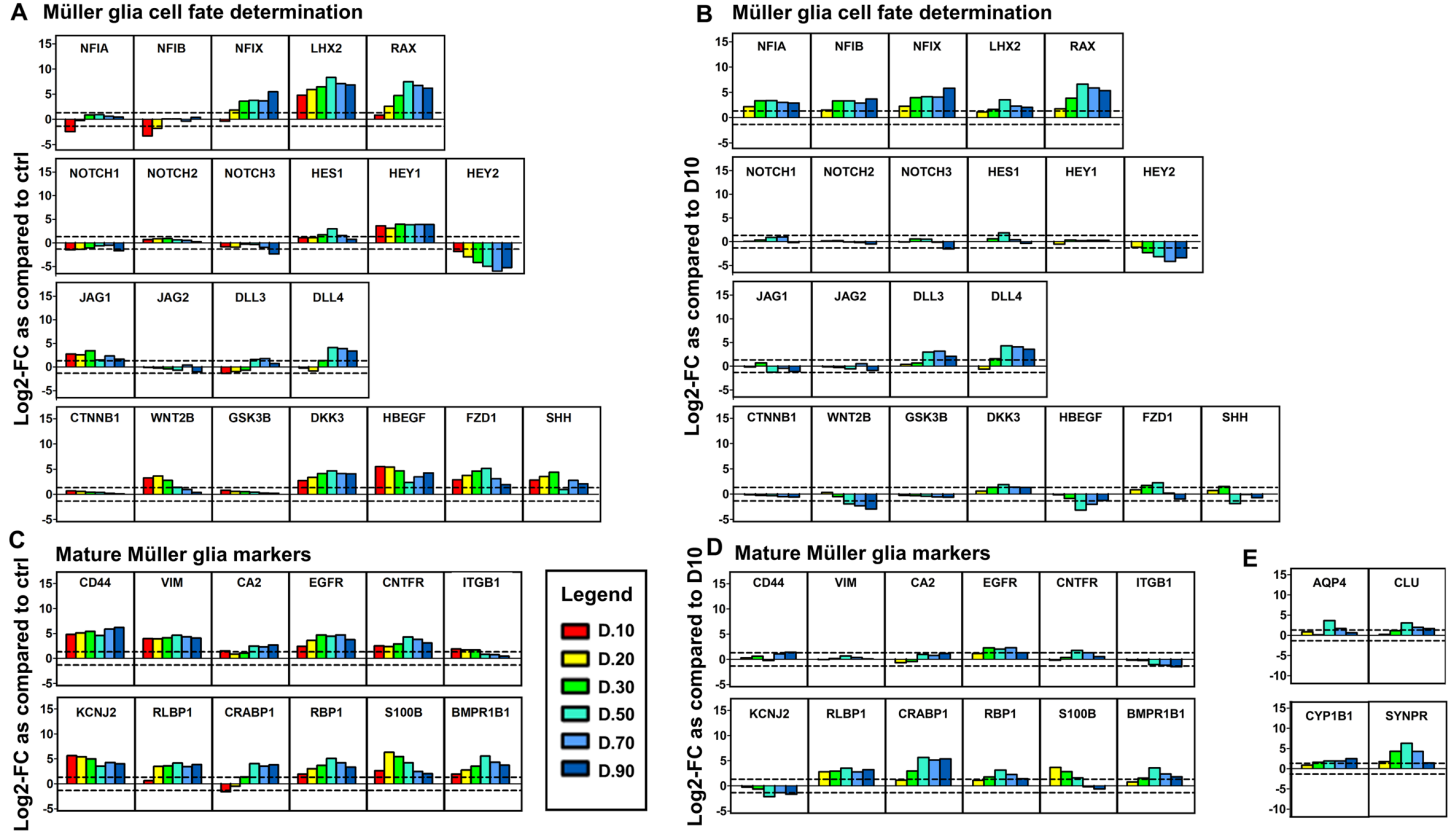
Examination of various genes expressed by Müller glia and selectively enriched in transcriptomics analysis. Bar graphs show log_2_Fold change (Log_2_FC) in selective gene expression for each CD29^+^/CD44^+^ sample as compared to undifferentiated stem cells (**A**/**C**) or cells isolated at D10 (**B**/**D**/**E**). Genes analysed comprise those associated with (**A**/**B**) Müller cell fate determination and (**C**/**D**/**E**) mature Müller glia markers. We regarded a 1.32 log2 fold change (increase and decrease) the cut off for significant change in expression (corresponding to fold changes of 2.5 and 0.4). Legend shows the age of the organoid from which the CD29^+^/CD44^+^ cells were isolated.

We first examined the expression of factors known to be involved in controlling Müller glia fate such as the NFI transcription factor family as well as the LHX2 and RAX genes^32,40,42^. *NFIA* and *NFIB* were downregulated in cells isolated at D10, and showed very little change in expression in isolated cells from D20–D90 as compared to the UD cells (Fig. 5A), however when comparing expression levels of D20- D90 cells to the D10 population, these genes were seen to be significantly increased (Fig. 5B). *NFIX* was upregulated in cells isolated at D20 and expression gradually increased in the CD29^+^/CD44^+^ cells isolated from organoids of increasing maturity as compared to UD stem cells (Fig. 5A). *LHX2* and *RAX* showed similar changes in expression. This pattern of expression was still observed when comparing D10 cells with D20–D90 cells (Fig. 5B).

Assessment of Notch signalling showed no change in expression of *NOTCH1*, *NOTCH2*, or *NOTCH3* in all cell preparations as compared to the UD (Fig. 5A) or the D10 cell population (Fig. 5B). *HES1* expression however, showed small fold-increases in expression in CD29^+^/CD44^+^ cells isolated at days 30, 50 and 70 after initiation of organoid differentiation as compared to the UD cells (Fig. 5A), but at only D30 as compared to D10 cells (Fig. 5B). *HEY1* was observed upregulated in the isolated cells and showed consistent expression in all samples examined as compared to UD (Fig. 5A). Expression of *HEY2* showed marked downregulation in CD29^+^/CD44^+^ cells at D10, and this trend was more prominent in cells isolated at D90 (Fig. 5A), this trend was mimicked when comparing to D10 isolated cells (Fig. 5B). Whilst *JAG1* showed a small decrease in their expression at D50 to D90 as compared to the starting material (Fig. 5A), DLL3 and DDL4 were slightly increased at these times points when compared to the UD cells. Comparing D20–D90 cells to the D10 population showed little difference in the expression of JAG1 or JAG2, but grandual increases in levels of DLL3 and DLL4 (Fig. 5B). Examination of genes coding for the WNT signalling pathway in CD29^+^/CD44^+^ cells also showed some changes in expression upon isolation at different stages of organoid maturation. Although *CTNNB1* and *GSK3B* expression in cells isolated between D10 and D30 did not show differences from UD stem cells, *WNT2B* was upregulated at this early stages. However its expression gradually decreased in cells isolated from D50 to 90 when compared with cells isolated at D10. *DKK3*, *HB-EGF* and *FZD1* genes showed increase in fold changes in cells isolated from retinal organoids at all stages of organoid differentaition (D10–90) as compared to the UD population (Fig. 5A). *SHH* which is also involved in retinal differentiation and gliogenesis, and can interact with WNT signalling showed increased expression in cells isolated from D10–30 retinal organoids, followed by a slight decrease between D50 and D70 (Fig. 5A). When examining these changes in gene expression from the D20–D90 CD29^+^/CD44^+^ cells as compared to the D10 cells, we observed little differences in expression over time (Fig. 5B).

The Müller glia markers *CD44* and *VIM* (vimentin) showed a marked increase and stable expression by CD29^+^/CD44^+^ cells isolated between D10 and D90 as compared to UD stem cells (Fig. 5C). *CA2* (carbonic anhydrase 2) showed little change in expression in cells isolated at early timepoints (D10–30), however its expression slightly increased in cells isolated from organoids at D50–90 as compared to the UD cells. *EGFR* (EGF receptor), *CNTFR* and *KCNJ2* (potassium Inwardly Rectifying Channel Subfamily J Member 2), were markedly upregulated as compared to the UD stem cells in cells isolated at all time points (D10–90), and showed little variance in expression (Fig. 5C). Conversely, if we compare expression levels from D20–D90 cells to the D10 isolated cells, there are few differences in the fold-changes of gene expression (*CD44*, *VIM*, *CA2*, *CNTFR*, *ITGB1*, *KCNJ2*; Fig. 5D). Mature Müller markers including, *RLBP1*, *CRALBP1* and *RBP1* showed higher increases in expression from the undifferentiated stem cells in cells isolated at later stages (from day 50) of organoid differentiation (Fig. 5C), as compared to the UD cells. Similary, this pattern reamined when comparing the gene expression in D20–D90 cells to the D10 cells (Fig. 5D), suggesting a shift in the function of these cells that aligns with the maturing organoid. *S100B* showed higher expression in cells isolated at ealier differentiation stages (D10, D20), whilst showing a gradual decline in cells upon increasing maturity (D30–D90) (Fig. 5C,D). *BMPR1B1* expression was upregulated in cells isolated at all timepoints (D10–90) but showed higher levels of expression in cells from D50–D90 organoids as compared to the UD cells or D10 cells (Fig. 5C,D). More interestingly, other classical Müller markers *AQP4*, *CLU*, *CYP1B1* and *SYNPR* which showed marked downregulation in D10–D90 isolated cells when compared to the UD stem cells (data not shown), showed marked increases when compared to the D10 cell population (Fig. 5E).

Furthermore, examination of the top 10 upregulated genes in each cell group (D10–D90) as compared to the UD stem cell population identified the Müller glia specific genes *RBP1* and *CYP1B1* and *CD44* to be amongst the highest in the D50, D70 and D90 cell populations. (Table SI2).

### Expression of transcripts coding for neurogenic and progenitor factors in CD29^+^/CD44^+^ cells isolated from retinal organoids at different stages of development and maturation

**Figure 6:**
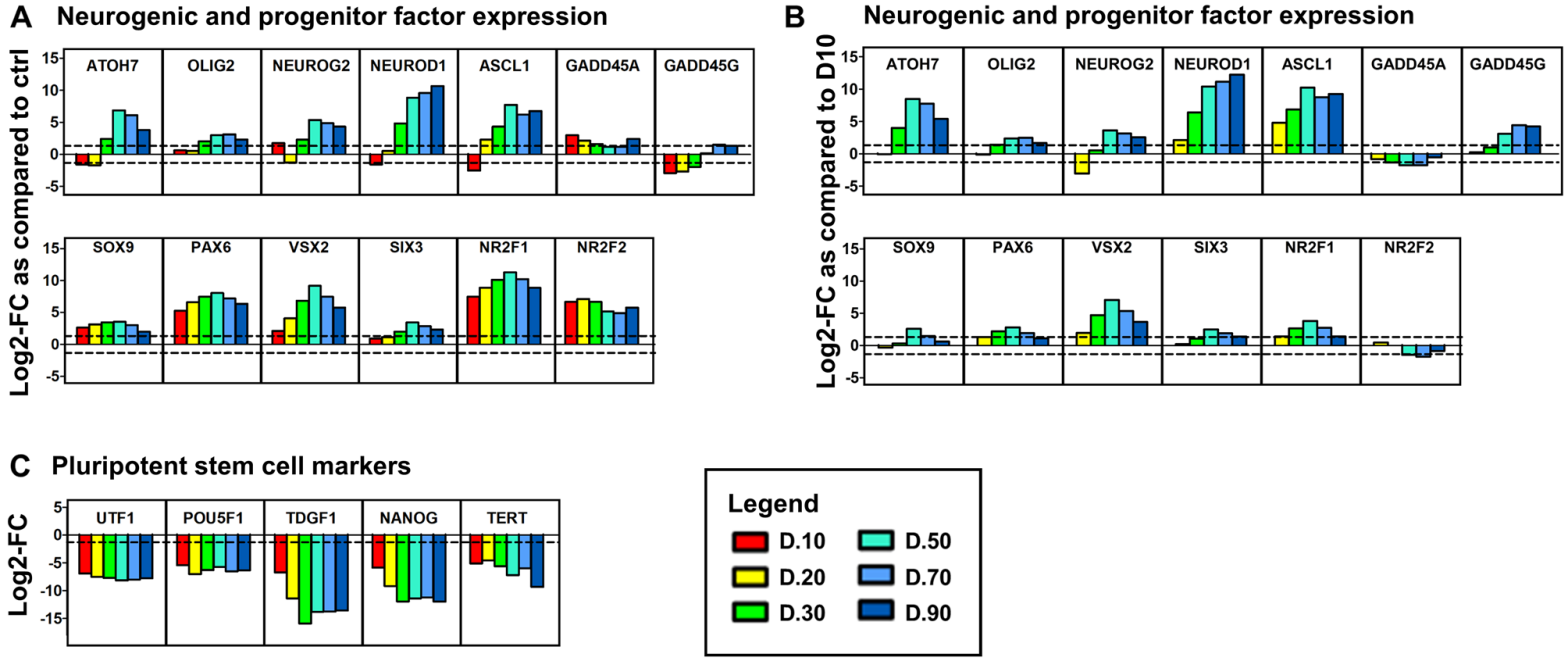
Examination of progenitor, and pluripotent gene expression. Bar graphs show log_2_Fold change (Log_2_FC) in selective gene expression for each CD29^+^/CD44^+^ cell sample as compared to undifferentiated stem cells (**A**/**C**) or as compared to the D10 isolated cells (**B**). Genes analysed comprise those associated with (**A**/**B**) neurogenic and progenitor factors and (**C**) pluripotent stem cell markers. We regarded a 1.32 log2 fold change (increase and decrease) the cut off for significant change in expression (corresponds to fold changes of 2.5 and 0.4). Legend shows the age of the organoid to which the CD29^+^/CD44^+^ cells were isolated.

Many of the neurogenic associated genes identified, including *ATOH7*, *OLIG2*, *NEUROG2*, *NEUROD1* and *ACL1*, showed major increases in expression in CD29^+^/CD44^+^ cells isolated from organoids after D30 as compared to the UD cells (Fig. 6A). These gene expression patterns were more pronounced when comparing D20–D90 isolated cells to the D10 population (Fig. 6B). Slight upregulation of *GADD45A* was observed in CD29^+^/CD44^+^cells isolated at D10, with similar levels being maintained up to D90 as compared to UD cells (Fig. 6A), however when comparing D20–D90 cells to the D10 isolated cells, there were no differences in expression levels (Fig. 6B). *GADD45G* was downregulated at D10–D30, with a very small pregulation occurring at D70 and D90, when compared with UD stem cells, but showed marked fold- increases in expression when comapring D50–D90 isolated cells to the D10 isolated cells (Fig. 6B). Fold-increases in the expression of the transcription factors *SOX9* and *PAX6* remained relatively stable in CD29^+^/CD44^+^ cells isolated at day 20 through to day 90. In contrast, *VSX2* and *SIX3* (also well documented to be involved in Müller glia proliferation and reprogramming), showed gradual increases in fold expression in cells isolated from organoids of increasing age (Fig. 6A). *NR2F1* and *NR2F2*, which are also associated with neurodevelopment, and which are highly expressed in retinal progenitors during retinogenesis were highly upregulated in CD29^+^/CD44^+^ cells isolated from organoids at all developmental stages examined (D10–90) as compared to the UD cells (Fig. 6A). Although these changes were not so pronounced when comparing D20–D90 isolated cells to the D10 population (Fig. 6B), gene expression patterns remained.

### Decreased expression of pluripotent stem cell markers indicates successful differentiation

To confirm differentiation of the embryonic stem cells, we analysed the log2-fold change in expression of pluripotent stem cell markers. All markers selected, which included *UTF1*, *POU5F1*, *TDGF1*, *NANOG* and *TERT* were highly downregulated in all CD29^+^/CD44^+^ cells isolated from retinal organoids as compared to UD stem cells (Fig. 6B).

### Validation of identified markers

**Figure 7:**
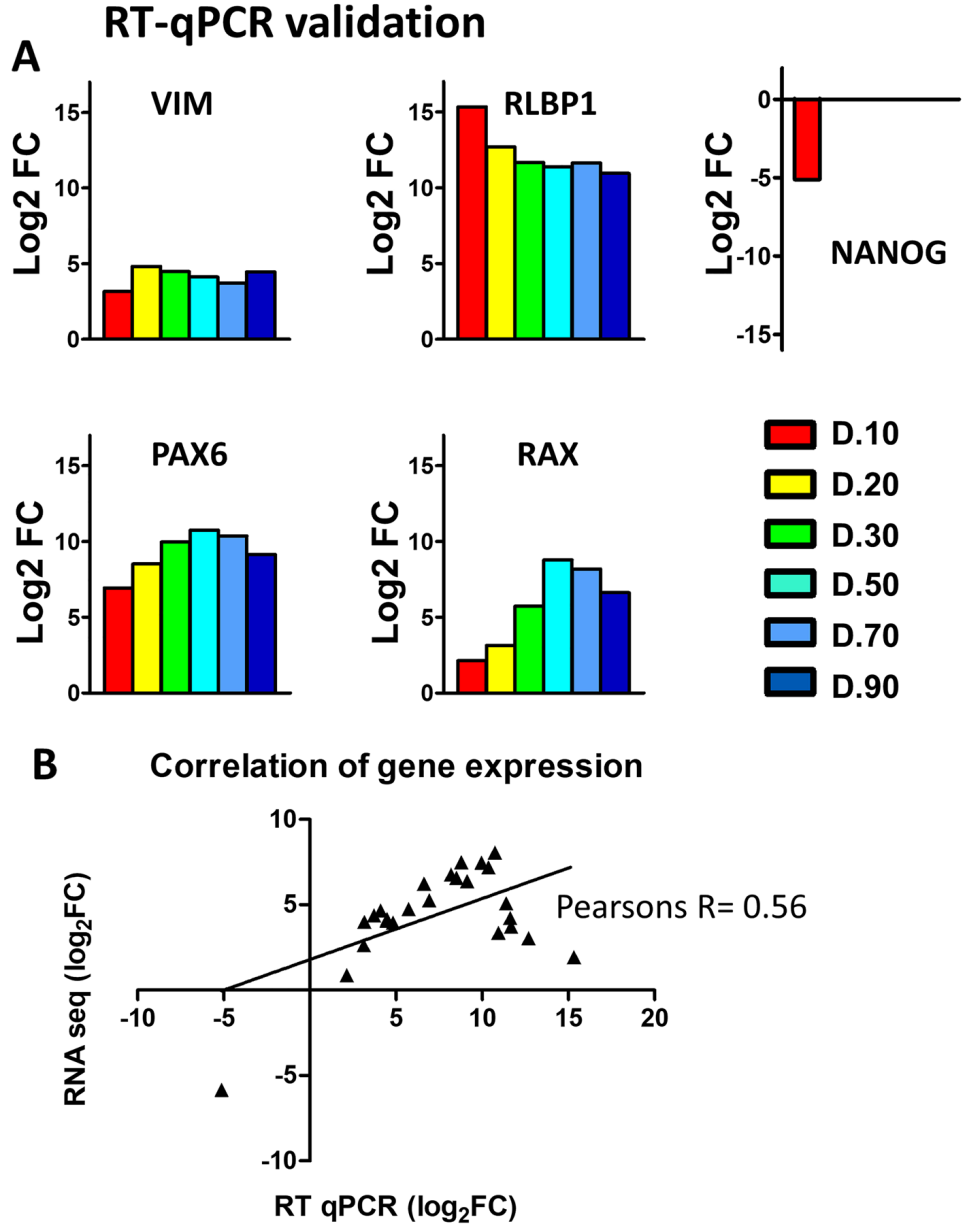
Validation of RNA seq analysis. (**A**) Bar graphs show log_2_ fold change of selected genes associated with Müller glia throughout the different stages of organoid formation and maturation. (**B**) The plot shows gene expression as log_2_ fold change as determined by qPCR and RNA-seq. The linear regression analysis and Pearson’s correlation co-efficient (Pearsons R = 0.56; R^2^ = 0.31; **P = 0.038) are shown.

Differentially expressed genes from RNA seq analysis were validated by qPCR using the same samples. Selected genes included the classical Müller glia markers *VIM*, *RLBP1*, as well as progenitor factors *PAX6*, and *RAX*, which showed similar fold changes in expression of mRNA levels between RNA seq and qPCR results (Fig. 7A). To confirm changes from the undifferentiated stem cells the pluripotent stem cell marker *NANOG*, showed marked downregulation in D10 isolated cells as compared to the UD cells. Expression of this gene was not detectable in samples extracted after D20 of organoid differentiation (Fig. 7A). Comparison of fold changes between RNA seq and qPCR data revealed significant correlation (Pearson’s R = 0.56; P = 0.0038; Fig. 7B).

## Discussion

Müller glia express the β1 integrin receptor CD29 and the cell surface glycoprotein CD44^43,44^, both of which are specific ligands for fibronectin. Expression of these surface proteins have facilitated the isolation of these cells from retina and retinal organoids formed by iPSC by selective adherence to fibronectin^33,43^. Our previous studies showed that using this protocol, > 97% of organoid dissociated cells adhered to this protein not only express CD29 and CD44 on their surface membrane^33^, but also express characteristic markers of Müller glia^33^. Previous studies in the literature have captured gene expression profiles in late stages of organoid development^25,28,30^, however there are still unanswered questions about the profile of these cells at earlier developmental stages. The current and previous studies from our group have suggested that Müller glia may constitute a progenitor population that arises much earlier in development that previously suggested. Therefore, the aim of our study was to profile the population of CD29^+^/CD44^+^ cells at earlier stages of organoid development in respect to their expression of Müller glia and progenitor markers.

The CD29^+^/CD44^+^ cells used in our study were isolated from retinal organoids and propagated for 3 days in culture to allow for selective binding to the ligand fibronectin. Due to the small numbers of cells produced by retinal organoids in culture it was not possible to conduct other cell selection methods such as fluorescence-activated cell sorting (FACS). Although some studies have reported changes in Müller glia identity upon adaptation to culture^45^, our previous and current investigations indicate that the profile of cells isolated from retinal organoids expressing CD29/CD44 remains stable, where we are able to expand these cells up to approximately 14 passages^33^. In addition, recent studies by Ning et al., 2022 and Couturier et al., 2021 have shown stable gene expression profile of Müller glia isolated from late stage (D90+) retinal organoids after several passages in culture^29,30^.

It has been traditionally accepted that specific genes are expressed by the so called early or late retinal progenitors, and most developmental studies have utilised such genes for in vivo genetic labelling to trace retinal development, or for examination of cell populations during development^40,46–49^. Müller glia are thought to be the last-born cells during ‘postnatal retinal development’ in vivo and share the same lineage with neurons, with multipotent retinal progenitors giving rise to both cell types^46,50^. However, it should be noted that earlier studies showing that Müller cells were the last cells to populate the retina, only examined the proliferation of retinal neurons and Müller glia in the early postnatal period^50^. Since at the time of birth a retina is present in all vertebrates, it would be important to question the nature of the cells forming the functionally immature retina at this stage. It can therefore be argued that tracing the proliferation of retinal cells in the postnatal eye does not provide conclusive evidence that Müller glia are the last cells to be born. Since retinal organoids are thought to recapitulate retinal development, they provide a tool in which we can re-examine Müller cell genesis. Much work has been undertaken in vitro regarding the development of photoreceptors in retinal organoids, but relatively few studies have primarily focused on the development of Müller glia, with most studies indicating that the appearance of Müller glia occurs at late stages of organoid development^25,51^. Sridhar et al. conducted a single cell transcriptomic comparison of hPSC derived retinal organoids and human foetal retina^28^ at various stages of development. Based upon expression of *RLBP1*, *AQP4* and *SLC1A3* to identify Müller glia, these markers were only detected in retina from foetal day 125, however expression of *SOX2* and *LHX2* were expressed relatively early in both foetal retina (FD59) and organoids (Day45) which may be an indication of progenitor like cells^28^. Similarly, other single cell transcriptomic investigations on whole organoids show expression of *RGR*, *RLBP1* and *APOE* which increased over the 18–38 wks investigated^25^. Recent studies have also isolated Müller glia from late-stage retinal organoids (D100+) with the aim of generating a transcriptomic profile^29,30^. Both studies isolated Müller glia showing stability over several passages, and expressing markers including *VIM*, *CLU*, *DKK3*, *SOX9*, *RLBP1*, *APOE*, *ITGB1*, *KCNJ1*, *FTL*, and *CRYAB*^29,30^. It is of interest that in the current study, we have isolated a population of CD29^+^/CD44^+^ cells at much earlier stages of retinal organoid development, that has shown classical Müller glia marker expression such as *VIM*, *CA2*, *RLBP1*, *CRABP1*, *KCNJ2* and *CD44* that remained relatively stable throughout isolation from D20–D90 retinal organoids. One of the markers considered to be characteristic of mature Müller cells is *CRALBP*. Our study supports this theory as a marked increase in expression was observed in cells isolated from D50 retinal organoids as compared to undifferentiated or those isolated from D10–30. This expression was maintained in cells isolated from D70–90 organoids. In addition, the top 10 upregulated genes in each cell population as compared to the UD stem cells, CD44, RLBP and CYP1B1 were present in D50–D90 cells (SI Tab2), suggesting that some maturity may be reached after just 50 days of organoid development.

However, we must also consider the developmental environment in which retinal organoids are formed in vitro. Whilst there are many advantages in using organoids in research, they lack characteristics associated with in vivo development such as vasculature, microglial cells as well as lack of exposure to visual stimuli, which may impact their transcriptomic profile. In addition, we may need to consider the variety of methods used by different laboratories to induce differentiation, which may influence timings of development and gene expression. We can therefore speculate that Müller glia in these models may not express some factors associated with visual stimuli such as potassium channels or glutamate processing to high levels. It would therefore be of interest to further develop these in vitro models to encompass some of these in vivo functions.

*NFIX* and *RAX* genes, from the NFI transcription factor family that regulates temporal patterning in retinal progenitor cells^40,52^, were significantly upregulated in cells isolated from D20 retinal organoids, when they are recognized to be in a relative immature state. Other genes, such as *LHX2*, which directly activates genes of the Notch pathway to induce glia proliferation and differentiation, and which is required for the differentiation and expression of Müller glia-specific markers such as glutamine synthetase^42,53^, was expressed in cells isolated at D10 and was gradually increased and maintained in CD29^+^/CD44^+^ cells isolated at D90. In this study, we noted a slight downregulation of *NOTCH1* and *NOTCH3*, and a marked decrease in *HEY2* expression as organoids progressed in maturation. In contrast, an increase in *HES1* and *HEY1* transcripts was observed upon initiation of organoid differentiation, and their levels of expression were maintained throughout the 90 day organoid culture. Notch signalling is important for retinal development as it promotes retinal progenitor proliferation and Müller glia differentiation^32,54,55^. The slight downregulation of *NOTCH1*, suggests that notch signalling may not be active in our isolated cells and is in agreement with reports that notch signalling is low in quiescent Müller glia^56^. Despite the downregulation in *NOTCH1* expression, increases in *HES1* and *HEY1* expression indicates some activation of the notch pathway, and these two genes have been implicated in the formation of Müller glia^32^. This is further supported by evidence that *HEY1* expression is associated with the maintenance of retinal progenitors^57^ as well as the regenerative response of Müller glia^58^. Furthermore, we showed that the notch ligands *JAG1* and *DLL4*, which are implicated in Müller glial cell differentiation^59^, were also upregulated D10 and D30, respectively, supporting the glial nature of CD29^+^/CD44^+^ cells. It is possible that differences in the expression of the notch signalling components are indicative of a dynamic development of Müller glia within the retinal organoid, and may allude to different timings of differentiation as well as different degrees of maturation of these cells. WNT pathway associated genes, including *WNT2B*, *DKK3* and *FZD1*, as well as *HBEGF* and *SHH*, were also upregulated in CD29^+^/CD44^+^ cells isolated from organoids at D10, with exception of *WNT2B* which gradually decreased in these cells upon increasing maturity, although it was still expressed at low levels at D90. Expression of these genes further support the nature of the cells analysed, as they are known to regulate Müller glia proliferation and differentiation^60–63^.

It is of interest that close similarities between Müller glia and multipotent late retinal progenitors have been widely reported^23,31,32,64,65^ yet, these are generally considered to be different cell populations, with Müller glia being proposed as a form of late stage retinal progenitor that gain specialized glial functions upon retinal maturation whilst retaining their late progenitor gene identity^31^. In the present study, classic genes recognized as markers of retinal progenitors including *ATOH7*, *OLIG2*, *NEUROG* 1 and 2 and *ASCL1*^65–67^, were also significantly upregulated in CD29^+^/CD44^+^ cells by D30 and their levels were gradually increased and maintained in cells isolated from organoids of increasing maturity up to D90. Other characteristic genes of retinal progenitors including *SOX9*, *PAX6*, *VSX2*, *SIX3*, and *NR2F1* and 2^6,24,53,68^ were upregulated from cells isolated at D10 of organoid differentiation, and their high levels maintained throughout the D90 study. Expression of genes coding for well-known markers of Müller glia, including *CD44*, *VIM*, *CA*2, *EGFR*, *ITGB1(CD29)*, *CNTFR*, *RBP1*, *S100B* and *BMPR1B1* were also significantly upregulated and maintained in CD29^+^/CD44^+^ cells from D10, with exception of *ITGB1* and *S100B*, which showed a downregulation as organoid maturation occurred, although they were still expressed at D90. Upregulation of RLBP1 a characteristic Müller glia marker was observed in cells from D20 and maintained until D90, whilst increase in the expression of *CRABP1*, another marker of mature Müller was only observed between cells isolated from D30 and D50 organoids. These are functional markers of Müller glia that mediate retinoid acid metabolism in the mature retina^69^ and therefore their increased expression may not be necessary until other neurons have developed in the retina. Genes often used to define mature Müller glia include *GLUL* (glutamine synthetase), *APOE* (apolipoprotein E), *RLBP1* (retinaldehyde binding protein 1), *KCNJ10* (Kir4.1*)*, and *CA2* (carbonic anhydrase 2)^24,31,70–72^. Interestingly, *CA2* was slightly upregulated in CD29^+^/CD44^+^ cells from D10 of organoid differentiation, and maintained at low levels throughout the study, whilst glutamine synthetase, which is only expressed by Müller glia in the neural retina^73^, and *APOE* were observed at basal levels in these cells throughout the study. As it is evident that glycogen and energy metabolism is not observed in Müller cells until later stages of development^18^, it is possible that these genes do not become upregulated and functional until retinal neurons fully mature in response to visual stimuli. This might explain the expression of genes ascribed to neural retinal development after birth. On this context, we have undertaken preliminary studies in which CD24^+^/CD44^+^ cells cultured for 24 h with glutamate have shown increase in the expression levels of *GLUL* and *CA2* transcripts (Supplementary Fig. 4), suggesting that synaptic release of this neurotransmitter may be needed to functionally activate GLUL and CA2, and this may reflect the low expression of some of these factors during early stages of retinal maturation.

To conclude, from the present observations it is not possible to identify differences between retinal progenitors and Müller glia within the CD29^+^/CD44^+^ cell population in retinal organoids formed by hPSC. They consistently appear to constitute a single cell type that express genetic features generally ascribed to both cell populations, and that are constantly observed throughout all stages of retinal development in vivo. Therefore, based on previous reports by others on the similarity between retinal progenitors and Müller glia, early ultrastructural studies of the developing retina in vivo^18–21^ and our current findings, we strongly propose that a population of cells with Müller glia characteristics emerges during initial stages of retinal organoid development, and that markers ascribed to Müller glia itself, may reflect the functional maturation of these cells upon development. Further studies would elucidate mechanisms that promote upregulation of genes that are required for the functional maturation of the Müller glia in the postnatal retina.

## Supplementary Information

Supplementary Table 1.

Supplementary Table 2.

Supplementary Figure 1.

Supplementary Figure 2.

Supplementary Figure 3.

Supplementary Figure 4.
