# Supplementary material for: Transcriptomics of CD29+/CD44+ cells isolated from hPSC retinal organoids reveals a single cell population with retinal progenitor and Müller glia characteristics
Source: Sci Rep. 2023 Mar 28;13:5081. doi: 10.1038/s41598-023-32058-w (PMC10050419; doi:10.1038/s41598-023-32058-w)
Supplement: Supplementary file 1 — Supplementary Table 1. [file 41598_2023_32058_MOESM1_ESM.docx]

| Gene | Primer |
| --- | --- |
| VIM | F- GGAAACTAATCTGGATTCACTC  R- CATCTCTAGTTTCAACCGTC |
| RLBP1 | F- CAAGTGGAAGAGAAGAACTTG  R- CAGAGTCCTTGGAAAAAGAAG |
| PAX6 | F- AGAGAATACCAACTCCATCAG  R- GATAATGGGTTCTCTCAAACTC |
| RAX | F- AGGCGGAAAAATAGAGTTTG  R- TACCCCAATATTCACTCCTC |
| NANOG | F- CCAGAACCAGAGAATGAAATC  R- TGGTGGTAGGAAGAGTAAAG |
|  |  |

**SI Tab 2. Human qPCR primers for SYBR Green assays**
