# Supplementary material for: Transcriptomics of CD29+/CD44+ cells isolated from hPSC retinal organoids reveals a single cell population with retinal progenitor and Müller glia characteristics
Source: Sci Rep. 2023 Mar 28;13:5081. doi: 10.1038/s41598-023-32058-w (PMC10050419; doi:10.1038/s41598-023-32058-w)
Supplement: Supplementary file 2 — Supplementary Table 2. [file 41598_2023_32058_MOESM2_ESM.docx]

| Day 10 vs UD | | |
| --- | --- | --- |
| gene ID | log2fold change | Adj. Pvalue |
| TERF1 | -3.82 | 3.8E-130 |
| FAM124A | -4.595 | 2.31E-79 |
| VASH2 | -5.23 | 3.99E-70 |
| RALGPS1 | -3.703 | 8.36E-66 |
| FRAT2 | -3.24 | 9.82E-57 |
| RABGAP1L | -3.719 | 7.39E-56 |
| SLC37A1 | -3.301 | 1.07E-55 |
| PIM2 | -3.514 | 7.47E-49 |
| CHEK2 | -2.666 | 1.58E-45 |
| DNMT3B | -5.296 | 4.61E-45 |

| Day 20 vs UD | | |
| --- | --- | --- |
| gene ID | log2fold change | Adj. Pvalue |
| TERF1 | -4.081 | 2.84E-150 |
| FAM124A | -4.756 | 4.01E-92 |
| VASH2 | -5.988 | 2.96E-91 |
| FRAT2 | -3.957 | 1.21E-83 |
| DNMT3B | -6.519 | 7.85E-68 |
| RALGPS1 | -3.341 | 1.57E-66 |
| PIM2 | -3.926 | 2.87E-61 |
| RABGAP1L | -3.866 | 5.24E-61 |
| ZNF788 | -3.174 | 3.22E-56 |
| AASS | -5.102 | 1.10E-50 |

| Day 30 vs UD | | |
| --- | --- | --- |
| gene ID | log2fold change | Adj. Pvalue |
| TERF1 | -3.832 | 3.92E-130 |
| FRAT2 | -3.811 | 6.50E-73 |
| FAM124A | -4.268 | 1.82E-71 |
| VASH2 | -5.178 | 1.34E-68 |
| DNMT3B | -6.336 | 7.56E-64 |
| ZNF788 | -3.565 | 1.32E-63 |
| PIM2 | -3.902 | 9.68E-59 |
| PHF20 | -1.53 | 1.49E-57 |
| UGP2 | -2.726 | 1.10E-53 |
| LIN28A | -9.767 | 3.13E-52 |

Top 10 downregulated genes

| Day 50 vs UD | | |
| --- | --- | --- |
| gene ID | log2fold change | Adj. Pvalue |
| TERF1 | -3.712 | 2.85E-124 |
| LIN28B | -3.918 | 1.46E-85 |
| DNMT3B | -7.055 | 8.47E-79 |
| CORO2A | -4.706 | 4.42E-71 |
| UGP2 | -3.123 | 2.24E-70 |
| SERPINB9 | -7.275 | 1.69E-66 |
| FAM46B | -4.372 | 1.44E-65 |
| FRAT2 | -3.415 | 9.51E-64 |
| PIM2 | -3.966 | 8.46E-62 |
| FAM124A | -3.655 | 2.98E-60 |

| Day 70 vs UD | | |
| --- | --- | --- |
| gene ID | log2fold change | Adj. Pvalue |
| TERF1 | -4.021 | 7.83E-144 |
| LIN28B | -4.987 | 7.96E-131 |
| DNMT3B | -6.826 | 7.25E-74 |
| UGP2 | -3.09 | 9.02E-69 |
| SERPINB9 | -7.275 | 6.91E-66 |
| FRAT2 | -3.471 | 1.54E-64 |
| LIN28A | -12.874 | 1.64E-64 |
| VASH2 | -4.933 | 9.61E-64 |
| FAM46B | -4.242 | 1.79E-61 |
| CECR1 | -5.332 | 6.94E-58 |

| Day 90 vs UD | | |
| --- | --- | --- |
| gene ID | log2fold change | Adj. Pvalue |
| LIN28B | -6.54 | 4.95E-181 |
| TERF1 | -4.053 | 5.64E-145 |
| DNMT3B | -8.1 | 1.63E-101 |
| VASH2 | -5.656 | 7.03E-80 |
| SERPINB9 | -8.221 | 5.51E-77 |
| FAM46B | -5.014 | 4.18E-75 |
| AASS | -6.054 | 1.63E-69 |
| FRAT2 | -3.609 | 1.53E-67 |
| UGP2 | -2.993 | 1.44E-64 |
| RCC2 | -2.386 | 2.37E-62 |

| Day 10 vs UD | | |
| --- | --- | --- |
| gene ID | log2fold change | Adj. Pvalue |
| CAPN2 | 4.972 | 1.7E-132 |
| EMP1 | 8.983 | 5.1E-125 |
| RHOBTB3 | 3.539 | 3.5E-116 |
| GLIPR1 | 8.12 | 4.8E-107 |
| TLR4 | 8.53 | 1.62E-90 |
| MSRB3 | 5.437 | 2.68E-84 |
| SFXN3 | 4.13 | 2.72E-79 |
| PHC2 | 4.523 | 1.82E-76 |
| PRSS23 | 7.046 | 1.41E-73 |
| FAM198B | 8.55 | 2.10E-71 |

| Day 20 vs UD | | |
| --- | --- | --- |
| gene ID | log2fold change | Adj. Pvalue |
| CAPN2 | 4.945 | 1.95E-131 |
| RHOBTB3 | 3.668 | 3.24E-125 |
| EMP1 | 8.72 | 7.62E-118 |
| GLIPR1 | 8.261 | 7.16E-111 |
| NT5E | 7.237 | 1.46E-84 |
| MSRB3 | 5.39 | 3.89E-83 |
| TLR4 | 8.137 | 1.68E-82 |
| MBNL2 | 5.948 | 8.13E-82 |
| SMPD1 | 3.384 | 3.21E-80 |
| PHC2 | 4.62 | 4.42E-80 |

| Day 30 vs UD | | |
| --- | --- | --- |
| gene ID | log2fold change | Adj. Pvalue |
| RHOBTB3 | 3.714 | 7.41E-128 |
| EMP1 | 8.869 | 8.48E-122 |
| CAPN2 | 4.708 | 3.73E-119 |
| GLIPR1 | 7.637 | 1.88E-94 |
| NT5E | 7.408 | 1.62E-88 |
| SMPD1 | 3.518 | 2.10E-85 |
| PHC2 | 4.519 | 3.31E-76 |
| MBNL2 | 5.719 | 3.68E-75 |
| SEC14L2 | 4.013 | 8.69E-74 |
| MSRB3 | 5.06 | 8.23E-73 |

Top 10 upregulated genes in each group.

| Day 50 vs UD | | |
| --- | --- | --- |
| gene ID | log2fold change | Adj. Pvalue |
| EMP1 | 8.518 | 4.77E-112 |
| RHOBTB3 | 3.229 | 2.91E-96 |
| SEC14L2 | 4.448 | 8.78E-92 |
| ZNF436 | 4.649 | 2.76E-85 |
| MBNL2 | 6.061 | 9.39E-85 |
| CAPN2 | 3.872 | 2.65E-80 |
| TBC1D9 | 6.389 | 1.21E-76 |
| RBP1 | 5.08 | 2.79E-76 |
| PHC2 | 4.485 | 2.65E-75 |
| DCBLD2 | 5.036 | 5.66E-74 |

| Day 70 vs UD | | |
| --- | --- | --- |
| gene ID | log2fold change | Adj. Pvalue |
| EMP1 | 8.564 | 1.98E-113 |
| NT5E | 7.81 | 1.98E-98 |
| SEC14L2 | 4.586 | 9.08E-98 |
| CAPN2 | 4.23 | 5.01E-96 |
| PHC2 | 4.855 | 3.02E-88 |
| RHOBTB3 | 3.013 | 8.62E-84 |
| CD44 | 5.879 | 1.60E-78 |
| MBNL2 | 5.709 | 4.99E-75 |
| GLIPR1 | 6.792 | 1.22E-74 |
| CYP1B1 | 6.614 | 8.31E-73 |

| Day 90 vs UD | | |
| --- | --- | --- |
| gene ID | log2fold change | Adj. Pvalue |
| RHOBTB3 | 3.346 | 2.28E-103 |
| EMP1 | 7.992 | 1.11E-98 |
| NT5E | 7.796 | 3.28E-98 |
| SEC14L2 | 4.576 | 3.23E-97 |
| GLIPR1 | 7.647 | 6.94E-95 |
| CD44 | 6.212 | 7.93E-88 |
| CYP1B1 | 7.234 | 3.32E-87 |
| CAPN2 | 3.986 | 2.87E-85 |
| PHC2 | 4.636 | 1.81E-80 |
| MBNL2 | 5.82 | 4.08E-78 |
