# Supplementary figures and images for: Transcriptomics of CD29+/CD44+ cells isolated from hPSC retinal organoids reveals a single cell population with retinal progenitor and Müller glia characteristics
Source: Sci Rep. 2023 Mar 28;13:5081. doi: 10.1038/s41598-023-32058-w (PMC10050419; doi:10.1038/s41598-023-32058-w)

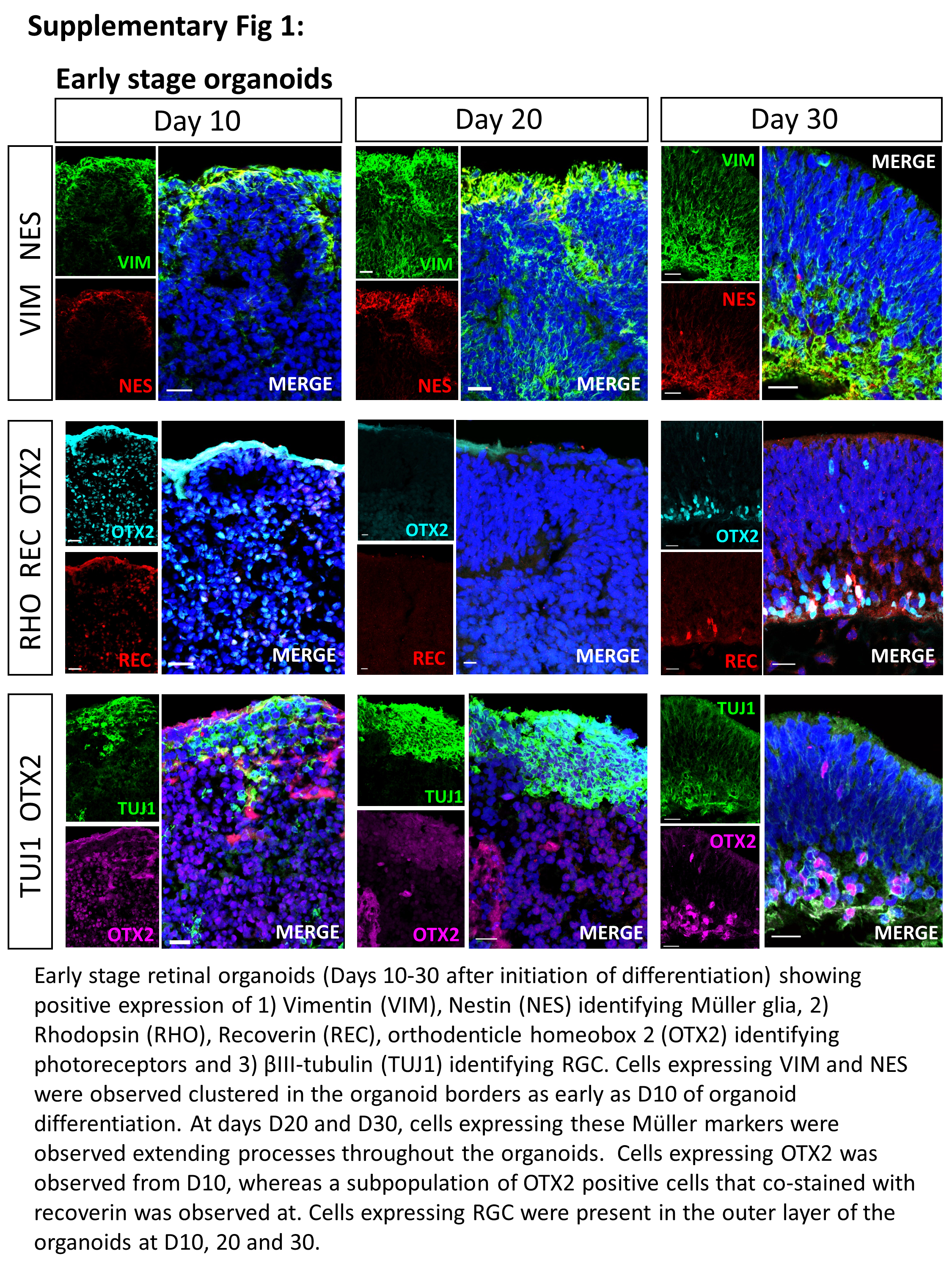

Supplement: Supplementary file 3 — Supplementary Figure 1. [file 41598_2023_32058_MOESM3_ESM.tif]

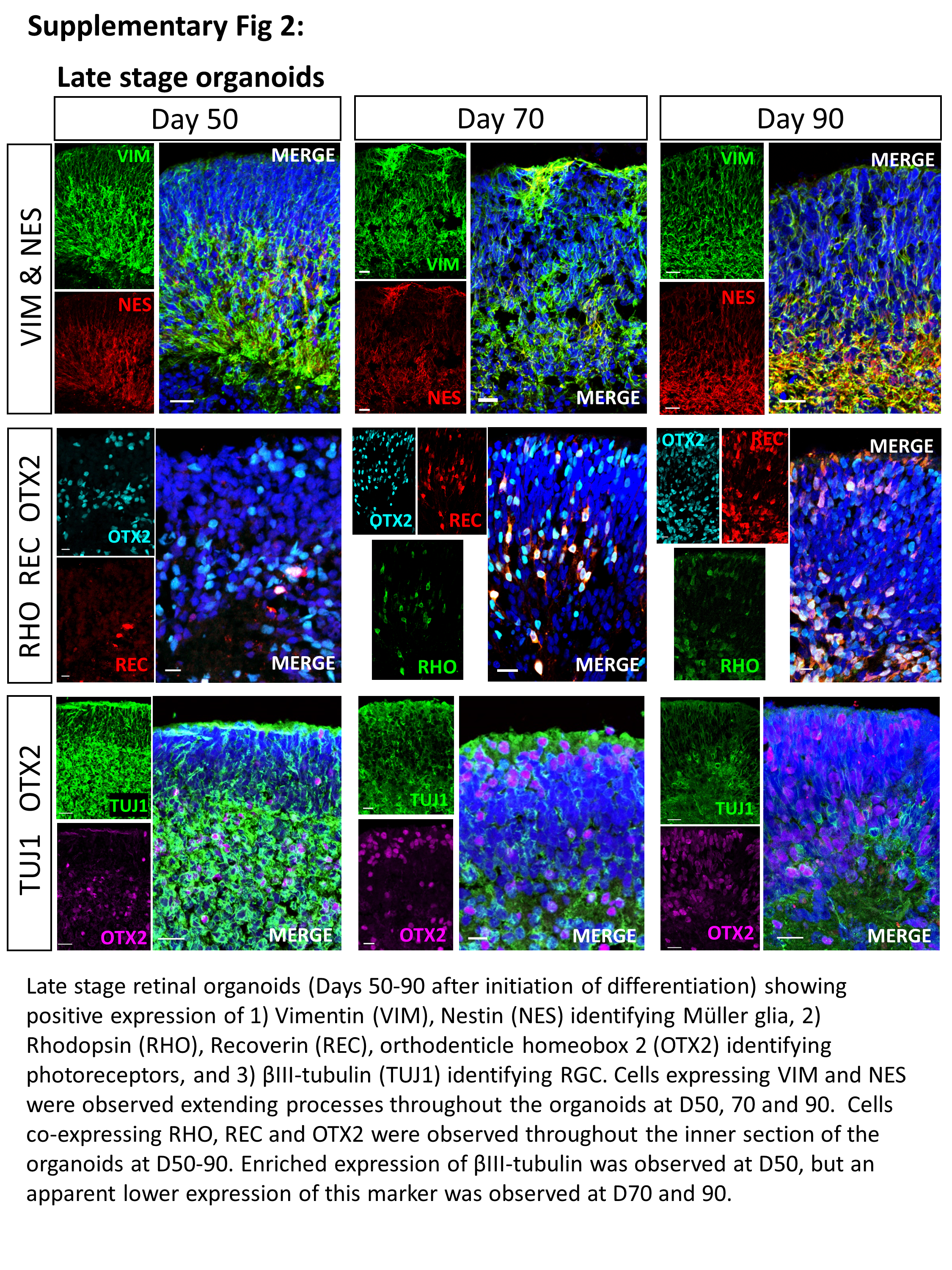

Supplement: Supplementary file 4 — Supplementary Figure 2. [file 41598_2023_32058_MOESM4_ESM.tif]

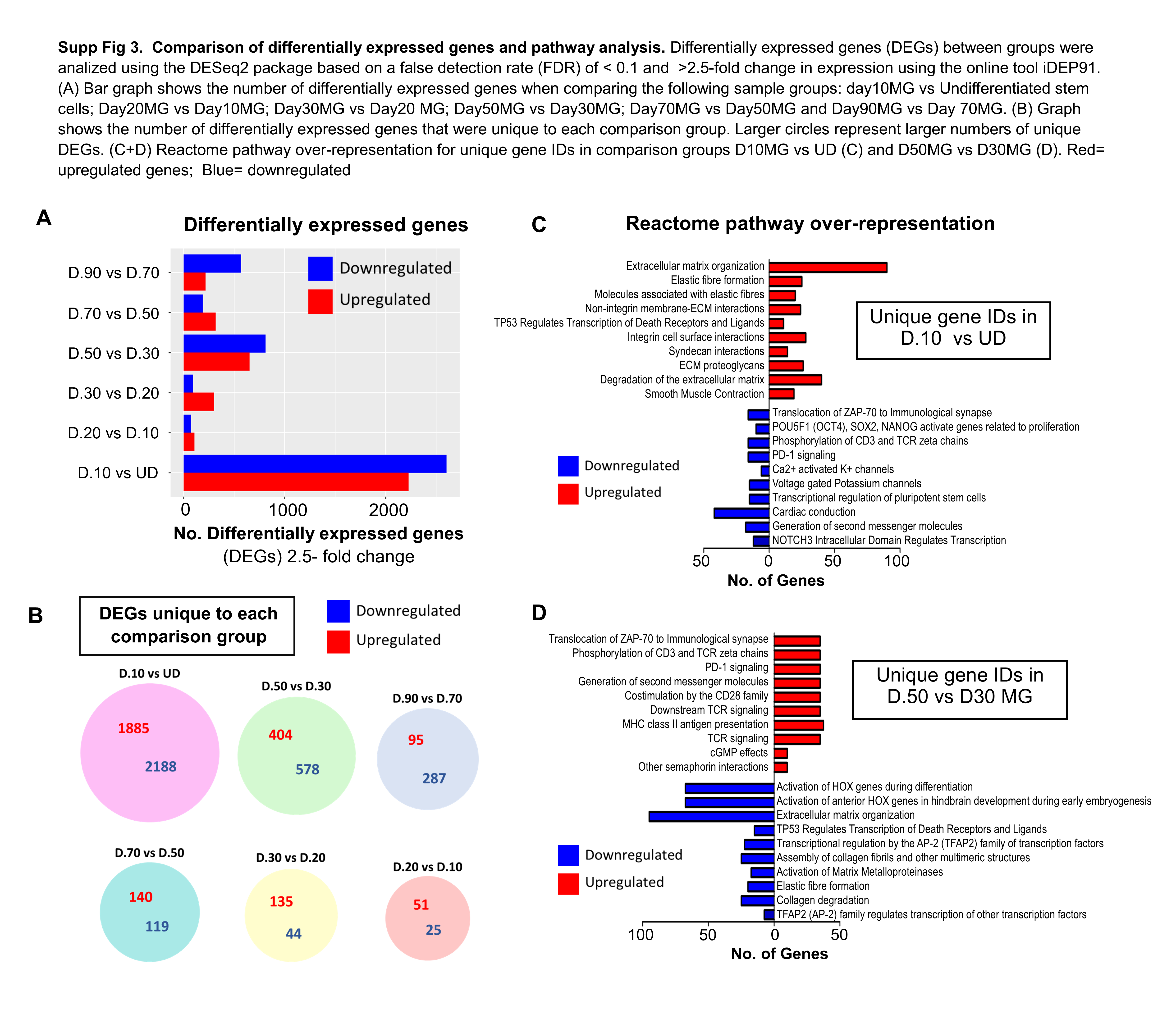

Supplement: Supplementary file 5 — Supplementary Figure 3. [file 41598_2023_32058_MOESM5_ESM.tif]

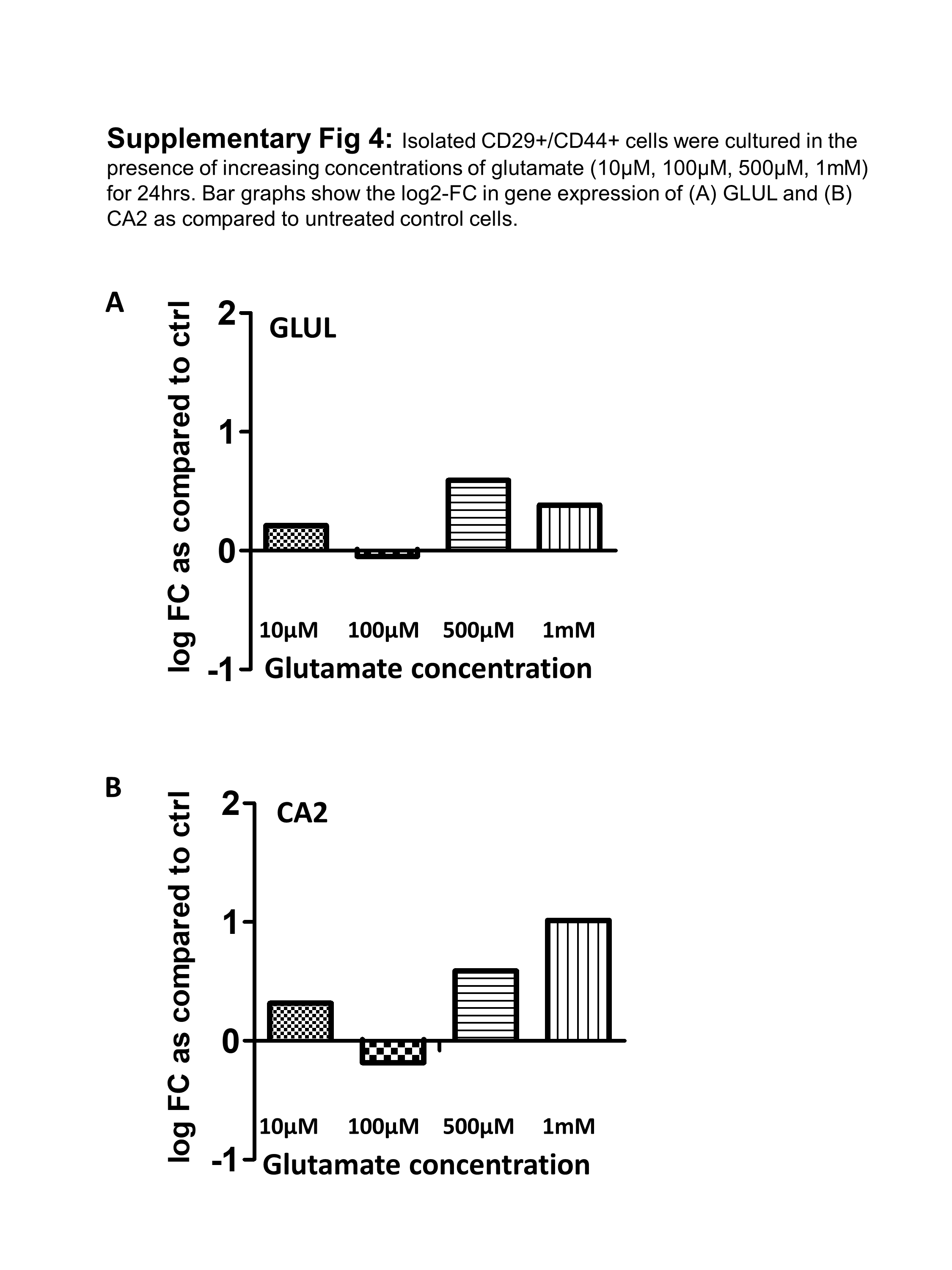

Supplement: Supplementary file 6 — Supplementary Figure 4. [file 41598_2023_32058_MOESM6_ESM.tif]
